# Does sex materially modulate responses to therapeutic hypothermia?

**DOI:** 10.1038/s41390-023-02624-z

**Published:** 2023-04-25

**Authors:** Kelly Q. Zhou, Joanne O. Davidson, Alistair J. Gunn

**Affiliations:** The Department of Physiology, The University of Auckland, Auckland, 1023 New Zealand

It is widely suggested in neonatal rodent studies that sex critically modulates the impact of many neuroprotective interventions, such as therapeutic hypothermia.^1^ Individual studies have reported variable outcomes but, overall, seem to show benefits for females but not for males. In part, this reflects the sheer variability of the commonly used approach, namely, the Rice–Vannucci model of unilateral carotid ligation and induced hypoxia. However, in a large retrospective meta-analysis of studies from a single laboratory using consistent methods over time, hypoxia–ischemia (HI) in postnatal day 7 (P7) rats followed by either normothermia (*n* = 305) or hypothermia (*n* = 317), there was significant hypothermic neuroprotection in females (*p* < 0.001) but not in males (*p* = 0.07).^1^

Moreover, there are striking sex differences in the cell death pathways activated after HI. In female rodent pups, cell death is largely dependent on ischemia-induced activation of caspases.^1,2^ By contrast, in males, cell death appears to be predominantly through the production of nitric oxide and activation of poly-ADP ribose polymerase 1, which leads to mitochondrial depolarization.^1,2^ Wood et al. concluded that it is possible that the different responses to therapeutic hypothermia seen between males and females could at least in part be attributed to the differential effect of cooling on these separate cell death pathways.

One obvious difference between males and females is the sex steroid hormones. A systematic review of progesterone or estradiol treatment after HI in neonatal rodents found a moderate-to-large effect of these hormones in reducing brain infarction and cell death, increasing neuronal survival, and reducing neuroinflammatory responses.^3^ Interestingly, the differences in cell death mechanisms were not solely due to sex hormones, as the differences persisted after ovariectomy in females.^2^

The evolution of hypoxic–ischemic brain injury over time may also be modulated by sex. In P10 mice exposed to the Rice–Vannucci model of HI, at day 1 after HI infarct size and seizure scores were not significantly different between males and females. However, by day 3, females had smaller infarct size and reduced behavioral deficits compared to males. In turn, males have increased microglial activation and heightened inflammatory responses than females. This suggests that the evolving differences in the immune response may contribute to differences in the degree of injury in males and females and their responses to different interventions.^4^

Stroke also shows marked sexual dimorphism in both preclinical and clinical studies.^2^ However, this is highly affected by age. In childhood and early adulthood, the risk of stroke is greater in males, and they have worse functional outcomes than females. Towards middle age, the incidence of stroke in females increase, coinciding with menopause and the reduction in female sex hormones. After middle age, the rates of stroke continue to increase in females and over the age of 85 years some studies report higher rates of stroke in females than in males.

In marked contrast to this literature, in the current issue Sewell et al. report that in a sub-group analysis of neonates (51 males, 50 females) with moderate to severe neonatal encephalopathy from the National Institute of Child Health and Human Development Induced Hypothermia (NICHD) trial, there were no differences in death or moderate-to-severe disability at 18–22 months’ corrected age between males and females (interaction *p* = 0.50) after whole-body cooling.^5^ This finding is similar to the lack of effect of sex (*p* = 0.606) after head cooling (44 males, 47 females) reported in a secondary analysis of the CoolCap trial.^6^ Thus, two well-powered randomized controlled trials have now reported no effect.^5,6^ Is this striking discordance simply a species difference, or could other factors contribute?

First, it is important to appreciate that in humans the magnitude of the difference in incidence and severity of neonatal encephalopathy between males and females is significant but modest such that it is only shown in very large, usually global studies.^7^ Thus, the findings of the NICHD and CoolCap studies should be taken to rule out a large difference in response but not smaller, more subtle sex-dependent differences in response. An individual patient meta-analysis will be needed to detect a small change.

Second, a potentially important confounding factor in the preclinical studies is that, on average, female rat pups have smaller body mass than males and smaller rodents are well known to have a greater reduction in temperature following anesthesia and anoxia.^8^ Typically, in rodent studies active cooling is only continued for 5 or 6 h. After this, the pups are returned to the nest and are usually not monitored for core temperature. During this time, endogenous hypothermia is very likely to occur.^9^ Thus, it is plausible that smaller rat pups could have lower temperatures due to greater heat loss secondary to their relatively greater surface area-to-volume ratios. This suggests that smaller rats, more often female, would have more prolonged lower “postcooling” temperatures and so have greater neuroprotection. Consistent with this hypothesis, in the Wood et al. meta-analysis, weight at the time of HI (P7) had a significant positive correlation with tissue area loss in hypothermia-treated rats,^1^ albeit this analysis was not segregated by sex.

Finally, studies in larger animals also do not support major sex differences. There were no sex differences in the response to asphyxia, resuscitation, and recovery in piglets.^10^ Similarly, in preterm fetal sheep there was no difference in the proportion of males and females that could not complete 25 min of umbilical cord occlusion because of asystole or mean arterial pressure falling below 8 mmHg.^11^ Intriguingly, in the fetuses in whom occlusion was stopped early, males and females showed different cardiovascular responses. Typically, studies of therapeutic hypothermia in piglets and fetal sheep have been underpowered to detect a sex difference^12^. Similarly to the clinical situation, a meta-analysis of therapeutic hypothermia studies in large animals after HI may be one way to assess whether there is an effect of sex.

Ultimately, even if there are subtle sex differences in human infants, these effects may not be sufficient to impact on survival and risk of moderate–severe disability. One reason for the lack of any obvious sex effects in humans and large animals may be that hypothermia suppresses a rather broad range of mechanisms of cell death^13^ and so offers correspondingly broad protection between boys and girls. Looking to the future, if the apparent sex differences in mechanisms of injury discussed above were to be validated in humans, this would raise the intriguing possibility that sex-specific add-on treatment strategies to hypothermia may be needed to achieve augmented neuroprotection.

## References

[1] Wood TR (2020). Variability and sex-dependence of hypothermic neuroprotection in a rat model of neonatal hypoxic-ischaemic brain injury: a single laboratory meta-analysis. Sci. Rep..

[2] Roy-O’Reilly M, McCullough LD (2018). Age and sex are critical factors in ischemic stroke pathology. Endocrinology.

[3] Durán-Carabali LE, Da Silva JL, Colucci ACM, Netto CA, De Fraga LS (2023). Protective effect of sex steroid hormones on morphological and cellular outcomes after neonatal hypoxia-ischemia: a meta-analysis of preclinical studies. Neurosci. Biobehav. Rev..

[4] Mirza MA, Ritzel R, Xu Y, McCullough LD, Liu F (2015). Sexually dimorphic outcomes and inflammatory responses in hypoxic-ischemic encephalopathy. J. Neuroinflammation.

[5] Sewell, E. K. et al. Evaluation of heterogeneity in effect of therapeutic hypothermia by sex among infants with neonatal encephalopathy. *Pediatr. Res*. 10.1038/s41390-023-02586-2 (2023). 10.1038/s41390-023-02586-2 PMC10843889 37012412

[6] Wyatt JS (2007). Determinants of outcomes after head cooling for neonatal encephalopathy. Pediatrics.

[7] Lee AC (2013). Intrapartum-related neonatal encephalopathy incidence and impairment at regional and global levels for 2010 with trends from 1990. Pediatr. Res..

[8] Sirimanne ES (1996). The effect of prolonged modification of cerebral temperature on outcome after hypoxic-ischemic brain injury in the infant rat. Pediatr. Res..

[9] Wood T (2018). Rectal temperature in the first five hours after hypoxia-ischaemia critically affects neuropathological outcomes in neonatal rats. Pediatr. Res..

[10] La Garde RP (2019). Sex differences between female and male newborn piglets during asphyxia, resuscitation, and recovery. Front. Pediatr..

[11] Bennet L (2007). Male disadvantage? Fetal sex and cardiovascular responses to asphyxia in preterm fetal sheep. Am. J. Physiol. Regul. Integr. Comp. Physiol..

[12] Draghi V (2019). Differential effects of slow rewarming after cerebral hypothermia on white matter recovery after global cerebral ischemia in near-term fetal sheep. Sci. Rep..

[13] Wassink G (2019). Therapeutic hypothermia in neonatal hypoxic-ischemic encephalopathy. Curr. Neurol. Neurosci. Rep..

